# Fluoride-Tolerant Mutants of *Aspergillus niger* Show Enhanced Phosphate Solubilization Capacity

**DOI:** 10.1371/journal.pone.0110246

**Published:** 2014-10-13

**Authors:** Ubiana de Cássia Silva, Gilberto de Oliveira Mendes, Nina Morena R. M. Silva, Josiane Leal Duarte, Ivo Ribeiro Silva, Marcos Rogério Tótola, Maurício Dutra Costa

**Affiliations:** 1 Departamento de Microbiologia, Universidade Federal de Viçosa, Viçosa, MG, Brasil; 2 Departamento de Solos, Universidade Federal de Viçosa, Viçosa, MG, Brasil; 3 Instituto de Ciências Agrárias, Universidade Federal de Uberlândia, Monte Carmelo, MG, Brasil; 4 Pesquisador Bolsista do Conselho Nacional de Desenvolvimento Científico e Tecnológico (CNPq), Brasília, DF, Brasil; CEA-Saclay, France

## Abstract

P-solubilizing microorganisms are a promising alternative for a sustainable use of P against a backdrop of depletion of high-grade rock phosphates (RPs). Nevertheless, toxic elements present in RPs, such as fluorine, can negatively affect microbial solubilization. Thus, this study aimed at selecting *Aspergillus niger* mutants efficient at P solubilization in the presence of fluoride (F^−^). The mutants were obtained by exposition of conidia to UV light followed by screening in a medium supplemented with Ca_3_(PO_4_)_2_ and F^−^. The mutant FS1-555 showed the highest solubilization in the presence of F^−^, releasing approximately 70% of the P contained in Ca_3_(PO_4_)_2_, a value 1.7 times higher than that obtained for the wild type (WT). The mutant FS1-331 showed improved ability of solubilizing fluorapatites, increasing the solubilization of Araxá, Catalão, and Patos RPs by 1.7, 1.6, and 2.5 times that of the WT, respectively. These mutants also grew better in the presence of F^−^, indicating that mutagenesis allowed the acquisition of F^−^ tolerance. Higher production of oxalic acid by FS1-331 correlated with its improved capacity for RP solubilization. This mutant represents a significant improvement and possess a high potential for application in solubilization systems with fluoride-rich phosphate sources.

## Introduction

Phosphate fertilizers are used intensively in agriculture for improving crop production. The use of low-reactivity rock phosphates (RPs), such as igneous RPs, combined with P-solubilizing microorganisms (PSM) has been shown to be an alternative for a sustainable use of P [1]–[4]. Several bacteria and fungi in the soil are able to solubilize P and to participate in the biogeochemical cycling of this element [5]. The ability of PSM to solubilize P is mainly associated with the release of metabolites with chelating or complexing properties, such as organic acids [3], [6]. The release of H^+^ during NH_4_^+^ assimilation and other metabolic processes that trigger H^+^ excretion are also reported as mechanisms of P solubilization [3], [7].

*Aspergillus niger* is a PSM with high P solubilization activity due to its capacity of medium acidification and production of organic acids with high metal complexation activity [3]. *Aspergillus niger* has been shown to solubilize either synthetic or natural apatites, i.e. RPs [3], [8]–[10]. Nevertheless, the chemical characteristics of RPs can interfere with the production of organic acids by PSM [4], [11], [12] and elements released during the solubilization may be toxic to microbial metabolism [4], [13]. Recently, it was demonstrated that F^−^ released from fluorapatite strongly inhibits RP solubilization by *A. niger* [4]. Fluoride also decreased fungal growth and the production of citric acid. Numerous other cellular processes can be negatively affected by F^−^, such as ion transport, secretion, endocytosis, gene expression, and, especially, enzymatic activity [14].

Given the ubiquitous distribution of F^−^ in RPs, most microbial RP solubilization systems studied so far have probably been operated under suboptimal conditions [4] and, thus, strategies to overcome the toxic effects of F^−^ on P solubilization must be developed. Such strategies might involve: i) solubilization systems in which only the microbial metabolites, and not the microorganism, are put into contact with RP [15]; ii) addition of adsorbents to remove F^−^ released during RP solubilization [16]; iii) isolation of strains naturally tolerant to F^−^ from environmental samples; and iv) mutagenesis of PSM to obtain mutants tolerant to F^−^.

Mutagenesis can be done by different strategies, such as the genetic engineering for the introduction of new information into the genome or deletion of chromosomal regions, induction of random mutations with physical and chemical mutagens, and manipulation of the sexual and parasexual cycles [17]. UV light is used for the genetic improvement of fungi [18]–[20]. The irradiation with UV light induces the formation of lesions on the DNA. The most common damages are the formation of cyclobutane pyrimidine dimers and the pyrimidine(6-4)pyrimidone photoproducts [21]. In the last few years, mutants of *Aspergillus tubingensis* [22] and *Penicillium rugulosum* [12] with increased P solubilization ability were obtained by random mutation with UV light. Thus, the objective of this work was to select *A. niger* mutants with increased P solubilization capacity in the presence of F^−^. Additionally, the effect of mutagenesis on fungal processes involved in P solubilization was also evaluated, namely the production of organic acids and medium acidification.

## Materials and Methods

### Microorganism and cultivation conditions

The strain *A. niger* FS1 was obtained from the Collection of Phosphate Solubilizing Fungi, Microbiology Department, Institute of Biotechnology Applied to Agriculture (BIOAGRO), Universidade Federal de Viçosa, Viçosa, MG, Brazil. Batch fermentations were performed in 125-mL Erlenmeyer flasks with 50 mL of the National Botanical Research Institute’s phosphate growth medium (NBRIP) [23] [5 g Ca_3_(PO_4_)_2_, 10 g glucose, 5 g MgCl_2_.6H_2_O, 0.25 g MgSO_4_.7H_2_O, 0.2 g KCl, 0.1 g (NH_4_)_2_SO_4_, 1 L deionized water]. Variations of this medium were obtained by replacing the P source or by supplementation with F^−^, as specified in each experiment. The medium pH was adjusted to 7.0 before the addition of the P sources. *Aspergillus niger* inoculum was added to flasks at the concentration of 10^6^ conidia from a suspension prepared in 0.1% (v/v) Tween 80. The flasks were incubated on an orbital shaker for 60 h at 32°C and 160 rpm [4]. Uninoculated flasks were used as controls.

### Mutagenesis of *A. niger* and screening of mutants for P solubilization in the presence of fluoride

Ten milliliters of a suspension of 10^6^ conidia mL^−1^, prepared in 0.1% (v/v) Tween 80, were irradiated for 16 min using a 13.8-W UV lamp (Mineralight) aiming at, approximately, 10% of survival. After UV irradiation, the conidia were spread onto Petri dishes (90×15 mm) containing potato dextrose agar (PDA) and incubated at 28°C for 48 h. The *A. niger* colonies obtained on PDA were then screened on solid NBRIP medium supplemented with NaF at 50 mg F^−^ L^−1^ (NBRIP-F). This concentration corresponds to the F^−^ amount that would be released from Araxá RP (3 g L^−1^) if all the F^−^ was made soluble [4]. The Petri dishes were incubated at 28°C for four days.

Colonies showing clear solubilization halos were transferred in triplicate to Petri dishes with NBRIP-F medium and incubated for six days at 28°C. After that, the diameters of the P solubilization halos of the mutants and the wild type (WT) were measured and compared. P-solubilizing ability was confirmed by quantification of soluble P (see analytical methods below) in liquid medium. Batch fermentations were done in liquid NBRIP-F medium and NBRIP medium with 3 g L^−1^ of Araxá RP (Table 1) as the only P source instead of Ca_3_(PO_4_)_2_. Inoculum preparation and cultivation conditions were as described above. Monosporic purification, successive cultivation on PDA and on NBRIP-F media was performed to ensure mitotic stability [24] of the *A. niger* mutants that showed the desired phenotype.

**Table 1:** Phosphorus and fluorine content and particle size of rock phosphates (RP).

RP	P (g kg^−1^)			F	Particle size
	Total	2% CA^a^	NAC^b^	(g kg^−1^)	(µm)
Araxá	139	19	5	16	<75
Catalão	162	21	4	22	<75
Itafós	39	8	-	9	<600
Patos de Minas	144	15	-	26	<75

a Soluble in 2% citric acid.

b Soluble in neutral ammonium citrate.

### Characterization of *A. niger* mutants

Mutants showing higher or lower P-solubilizing ability than the WT were selected for further studies. The mutants were cultivated in NBRIP containing a soluble P source (1 g L^−1^ K_2_HPO_4_) instead of Ca_3_(PO_4_)_2_, without or with F^−^ (50 mg L^−1^), to allow the evaluation of the mutagenesis effects on the production of organic acids and fungal growth. The flasks were incubated on an orbital shaker for 60 h at 32°C and 160 rpm. The experiments were performed in triplicate following a completely randomized design (CRD).

The effect of mutagenesis on P solubilization in the presence of F^−^ was also investigated. For this, the selected mutants were grown in liquid NBRIP-F medium and NBRIP with Araxá RP (3 g L^−1^) as the only P source. NBRIP medium without F^−^ was used as a control. The experiment was performed in triplicate under a CRD. At the end of incubation, solubilized P, dry biomass, pH, titratable acidity, and the concentration of organic acids were analyzed (see analytical methods below).

### Effect of fluoride on RP solubilization by *A. niger* mutants

The mutants and the WT were grown on NBRIP medium with Araxá RP as the only P source and supplemented with increasing concentrations of F^−^, ranging from 0 to 50 mg L^−1^ at intervals of 5 mg L^−1^. At the end of the experiment, solubilized P, fungal biomass, Y_P/B_ [P/biomass yield = Solubilized P (mg)/dry biomass (g), in 50 mL of medium] [3], and pH were determined. Treatments were arranged in a CRD with three replications at the central point. The results were submitted to regression analyses.

### Solubilization of different P sources by *A. niger* mutants

The mutants with the highest P solubilization activity were also tested with other P sources besides Araxá RP. Pure P sources, namely AlPO_4_ and FePO_4_, were added to NBRIP medium [without Ca_3_(PO_4_)_2_] at an equivalent concentration of 1 g P L^−1^. The RPs evaluated were Catalão, Patos de Minas, and Itafós (Table 1). Due to the high variability in the P and F content, all the P-bearing rocks were added at 3 g L^−1^. At the end of the experiment, soluble P and fungal biomass were determined (see analytical methods below). The experiment was conducted under a CRD with three replications.

### Analytical methods

After incubation, the spent media were centrifuged at 5,000 *g* for 20 min and filtered through quantitative filter paper (8-µm pores). The filtrate was used to determine solubilized P, pH, titratable acidity, and organic acids. Solubilized P was quantified spectrophotometrically by an ascorbic acid method [25]. Titratable acidity was measured by titrating 5 mL of the culture filtrate to pH 7 with 0.1 M NaOH using bromothymol blue as a pH indicator. The fungal biomass retained on the filter paper was collected, dried in an oven at 70°C to constant weight, and incinerated at 500°C for 8 h. Biomass yield was determined by subtracting the weight of the residue left after incineration from the weight of the dried fungal mycelium. This method avoids the overestimation of fungal biomass by the adherence of phosphate particles on the mycelium [24].

Based on previous results for the isolate *A. niger* FS1, organic acid analyses were focused on citric, gluconic, and oxalic acids [3]. For this, the culture filtrate was further passed through 0.22-µm nylon filters. Citric and gluconic acids were determined by ultra-performance liquid chromatography-tandem mass spectrometry (UPLC/MS/MS) using a UPLC Agilent 1290 Series coupled to a 6400 Triple Quadrupole mass spectrometer. Chromatographic separations were carried out using an Agilent ZORBAX Eclipse Plus C18 column (1.8 µm, 2.1 mm×50 mm). The column temperature was controlled at 35°C. An isocratic flow of 97% water and 3% acetonitrile at a flow rate of 0.45 mL min^−1^ was used. The sample injection volume was 10 or 20 µL according to the acid concentration in each sample. The eluate from UPLC was introduced into MS through an APCI source in negative mode. The acids were identified in an analysis time of 1.5 min using multiple reaction monitoring (MRM). The transitions 191.18 to 110.90 and 195.15 to 128.93 were monitored to citric and gluconic acids, respectively, according to standards (Sigma Chemical Co. St. Louis, MO). For oxalic acid analyses, to ensure the solubilization of calcium oxalate precipitates formed during the solubilization process, the culture supernatants were acidified with 37% HCl to a pH value of approximately 0.5 before filtration [4]. The compound was determined using an Ultimate Dionex 3000 HPLC equipped with a refraction index (RI) detector. The chromatographic separation was carried out in a Rezex ROA-Organic Acid H^+^ (8%) column (8 µm, 300 mm×7.8 mm) with sample injection volume of 20 µL and analysis time of 15 min. The mobile phase corresponded to sulfuric acid (5 mmol L^−1^) with a flow rate of 0.7 mL min^−1^. Oxalic acid was quantified by reference to the peak areas obtained with appropriate standards (Merck, Germany).

### Statistical analyses

The data were subjected to ANOVA and multiple mean comparisons were performed through the Tukey or Scott-Knott tests (*P*<0.05) using the statistical software Minitab 16 and Sisvar.

## Results

### Mutagenesis of *A. niger*

Twenty-nine mutants showing higher and lower solubilization halos than the WT were obtained in NBRIP-F medium after exposure to UV light (data not shown). These mutants were tested in liquid medium to quantify the solubilization of Araxá RP and Ca_3_(PO_4_)_2_ supplemented with F^−^ (Table 2). Based on these data, three strains were selected for further studies according to the following criteria: FS1-555 showed the highest increase in P solubilization from Ca_3_(PO_4_)_2_ with F^−^ (67%); FS1-331 showed the highest increase in P solubilization from Araxá RP (64%) and FS1-375 showed decreased solubilized P for both sources. The mutants FS1-55 and FS1-42 also showed reduced ability to solubilize P, but were not chosen for further analyses because of their substantial growth decrease (Table 2).

**Table 2:** Solubilized P and biomass produced by *Aspergillus niger* FS1 mutants compared to the wild type.

	NBRIP-F			NBRIP with Araxá RP		
Strains	Soluble P (mg L^−1^)	I or D (%)^a^	Dry biomass (mg flask^−1^)	Soluble P (mg L^−1^)	I or D (%)	Dry biomass (mg flask^−1^)
WT	346.1 b	-	42.7 b	61.1 d	-	45.6 b
FS1-555	554.8 a	67	34.9 c	85.3 c	42	28.7 e
FS1-326	487.9 a	45	8.0 g	40.4 e	–41	30.0 e
FS1-307	458.5 a	36	45.1 b	69.2 d	14	36.2 c
FS1-512	435.4 b	29	21.4 f	75.7 c	25	26.6 f
FS1-506	429.5 b	23	30.5 d	49.4 d	–15	25.5 f
FS1-261	424.5 b	21	46.3 b	67.2 c	8	26.5 f
FS1-408	416.8 b	62	5.9 g	86.2 c	50	21.2 f
FS1-440	406.9 b	19	39.1 c	101.2 b	51	44.5 b
FS1-442	403.9 b	16	36.6 c	78.1 b	22	23.3 f
FS1-270	400.9 b	48	8.6 g	104.1 a	55	29.8 e
FS1-347	395.8 b	13	9.9 g	37.3 e	–30	13.5 g
FS1-250	392.7 b	9	51.6 a	77.2 c	30	46.4 a
FS1-331	391.2 b	12	24.2 f	110.9 a	64	17.2 g
FS1-262	372.6 b	5	46.9 b	69.9 d	16	48.3 a
FS1-98	370.6 b	7	42.6 b	50.7 d	–13	33.5 c
FS1-8	368.1 b	6	44.7 b	74.6 c	17	33.3 c
FS1-22	366.6 b	6	42.7 b	43.3 e	–23	33.0 d
FS1-406	360.8 b	4	22.0 f	67.4 d	10	23.3 f
FS1-164	339.9 c	–2	27.0 e	53.3 d	–12	38.4 c
FS1-537	334.9 c	–4	34.8 c	96.3 b	61	41.9 b
FS1-48	303.0 c	–11	23.3 f	75.0 c	22	35.3 c
FS1-166	287.1 c	–15	30.7 d	54.7 d	–10	47.3 a
FS1-28	285.8 c	–15	28.5 e	57.9 d	–5	34.7 c
FS1-41	284.7 c	–15	37.0 c	55.9 d	–8	43.7 b
FS1-123	242.5 c	–33	47.6 b	55.1 d	–10	49.8 a
FS1-110	212.5 c	–25	52.4 a	58.1 d	–6	40.6 b
FS1-375	196.2 c	–48	20.1 f	30.8 e	–52	43.6 b
FS1-55	120.8 d	–56	18.0 f	14.3 f	–72	7.7 h
FS1-42	59.5 d	–71	1.0 h	33.4 e	–43	12.7 g

The experiments were carried out in liquid NBRIP medium supplemented with F^−^ at 50 mg L^−1^ (NBRIP-F) or NBRIP with 3 g L^−1^ of Araxá RP as the only P source. Flasks were incubated for 60 h at 32°C and 160 rpm.

a The percent increase or decrease of solubilized P of the mutants was calculated based on the solubilized P by the WT in the media NBRIP-F and NBRIP with RP, respectively 346 and 61 mg L^−1^. P (%) = (mMUT–mWT)/mWT×100; mMUT: mean solubilized P for the mutant; mWT: mean solubilized P for the wild type. Means followed by the same letter are not significantly different according to the Scott Knott test (*P<*0.05).

### Characterization of *A. niger* mutants

**Figure 1:**
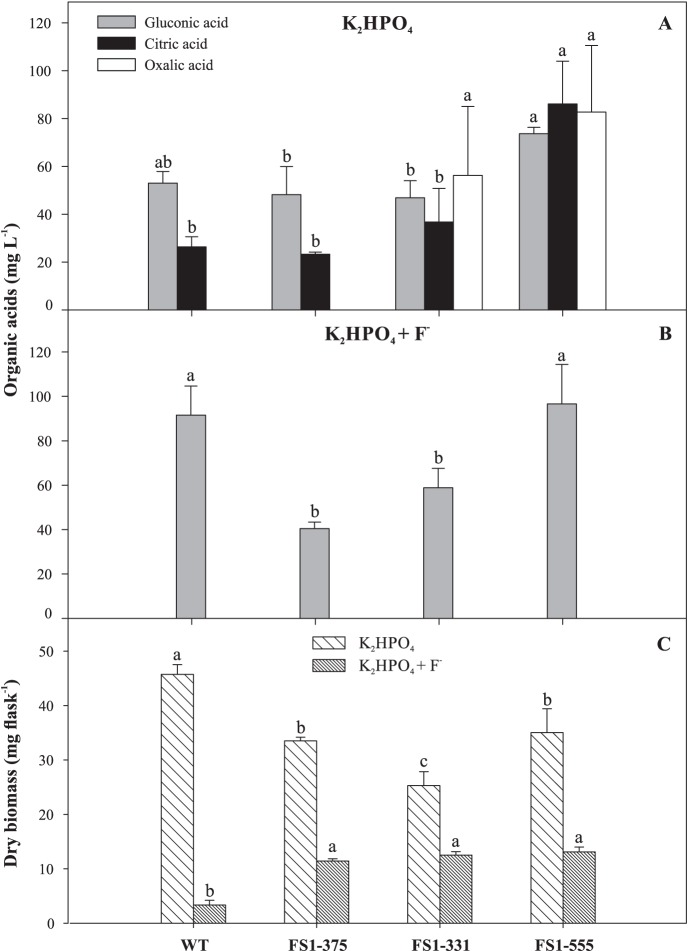
Organic acids produced by *Aspergillus niger* FS1 mutants and the wild type grown on NBRIP medium with K_2_HPO_4_ (1 g L^−1^) as the P source (A) and supplemented with fluoride (50 mg L^−1^) (B); and fungal dry biomass produced in both conditions (C). The experiment was incubated for 60 h at 32°C and 160 rpm. For each cultivation condition, columns with the same letter are not significantly different by the Tukey’s test (*P<*0.05). Error bars represent the mean standard deviation (n = 3).

Mutagenesis and F^−^ altered the profile of organic acid production by the strains (Fig. 1a, b). Only the mutants FS1-331 and FS1-555 produced oxalic acid in the medium with K_2_HPO_4_ (Fig. 1a). Citric and gluconic acids were produced by all the strains (Fig. 1a). The addition of F^−^ inhibited the production of citric and oxalic acids (Fig. 1b). F^−^ also decreased fungal growth, however, the mutants were more tolerant to it than the WT (Fig. 1c).

The mutant FS1-331 showed the highest value of solubilized P when grown in the presence of Araxá RP, solubilizing 70% more P than the WT (Table 3). The FS1-555 also increased the solubilization of Araxá RP by 15% compared to the WT. The titratable acidity was higher in the treatments inoculated with the mutants FS1-331 and FS1-555 on Araxá RP. In the medium with Ca_3_(PO_4_)_2_+F^−^, the mutant FS1-555 was the most effective at P solubilization. Moreover, in the media inoculated with this mutant, the lowest pH values were observed. In the medium with Ca_3_(PO_4_)_2_, no difference was observed between the values of solubilized P by the mutants FS1-331 and FS1-555 and the WT. A significant decrease in P-solubilization and higher pH values were observed for the mutant FS1-375 in the three media evaluated. The biomass of FS1-375 was also lower than that of the WT for all the P sources tested.

**Table 3:** Solubilized P, dry biomass, pH and titratable acidity in NBRIP medium with Araxá RP (3 g L^−1^) as the only P source, Ca_3_(PO_4_)_2_ (5 g L^−1^) + F^−^ (50 mg L^−1^), or Ca_3_(PO_4_)_2_ (5 g L^−1^) after the cultivation of *Aspergillus niger* FS1 mutants and the wild type for 60 h at 32°C and 160 rpm.

Strains	Solubilized P (mg L^−1^)	Dry biomass (mg flask^−1^)	pH	Titratable acidity (mmol H^+^ L^−1^)
**Araxá RP**				
**WT** a	60.70 c	41.63 a	2.91 b	2.5 bc
**FS1-375** b	33.32 d	34.17 b	3.27 a	0.8 c
**FS1-331**	102.75 a	26.43 c	2.79 b	6.4 a
**FS1-555**	69.66 b	28.73 c	2.77 b	6.0 ab
**Ca_3_(PO_4_)_2_+ F^−^**				
**WT**	382.81 b	35.87 a	3.49 a	13.0 ab
**FS1-375**	199.58 c	16.07 d	3.46 a	4.7 b
**FS1-331**	372.25 b	20.73 c	3.20 ab	12.5 ab
**FS1-555**	558.90 a	29 b	3.07 b	17.1 a
**Ca_3_(PO_4_)_2_**				
**WT**	766.31 a	51.17 a	2.86 b	25.2 a
**FS1-375**	458.68 b	23.43 b	3.45 a	13.9 a
**FS1-331**	694.54 a	32.63 b	3.08 b	16.7 a
**FS1-555**	744.82 a	52.63 a	3.47 a	14.8 a

Means followed by the same letter are not significantly different according to the Tukey’s test (*P<*0.05).

a WT: *Aspergillus niger* FS1 wild type.

b Mutant with a significant decrease in P solubilization potential (negative mutant).

The production of the citric and oxalic acids differed among the mutants and the WT (Table 4). The mutants FS1-331 and FS1-555 were the only ones that produced oxalic acid in the presence of Araxá RP. All the mutants produced less citric acid than the WT, while the production of gluconic acid was similar for all strains. Only gluconic and citric acids were detected in the medium with Ca_3_(PO_4_)_2_+F^−^, and the mutant FS1-555 produced the highest amount of gluconic acid. The mutant FS1-375 produced the lowest amounts of organic acids in the three media evaluated.

**Table 4:** Organic acids (mg L^−1^) produced by *Aspergillus niger* FS1 mutants and the wild type in NBRIP medium with Araxá RP (3 g L^−1^) as the only P source, Ca_3_(PO_4_)_2_ (5 g L^−1^) + F^−^ (50 mg L^−1^), or Ca_3_(PO_4_)_2_ (5 g L^−1^) after 60 h of incubation at 32°C and 160 rpm.

Strains	Gluconic acid	Citric acid	Oxalic acid
**Araxá RP**			
**WT**	287 a	164 a	nd
**FS1-375**	190 a	100 c	nd
**FS1-331**	225 a	126 b	27 a
**FS1-555**	240 a	135 b	22 b
**Ca_3_(PO_4_)_2_+ F^−^**			
**WT**	1855 b	455 b	nd
**FS1-375**	1281 c	103 c	nd
**FS1-331**	1013 c	711 a	nd
**FS1-555**	2482 a	250 bc	nd
**Ca_3_(PO_4_)_2_**			
**WT**	4676 c	1114 a	nd
**FS1-375**	5930 b	197 c	nd
**FS1-331**	7655 a	629 b	nd
**FS1-555**	6382 ab	618 b	nd

Means followed by the same letter are not significantly different according to the Tukey’s test (*P<*0.05).

nd: not detected.

### Effect of fluoride on RP solubilization by *A. niger* mutants

**Figure 2:**
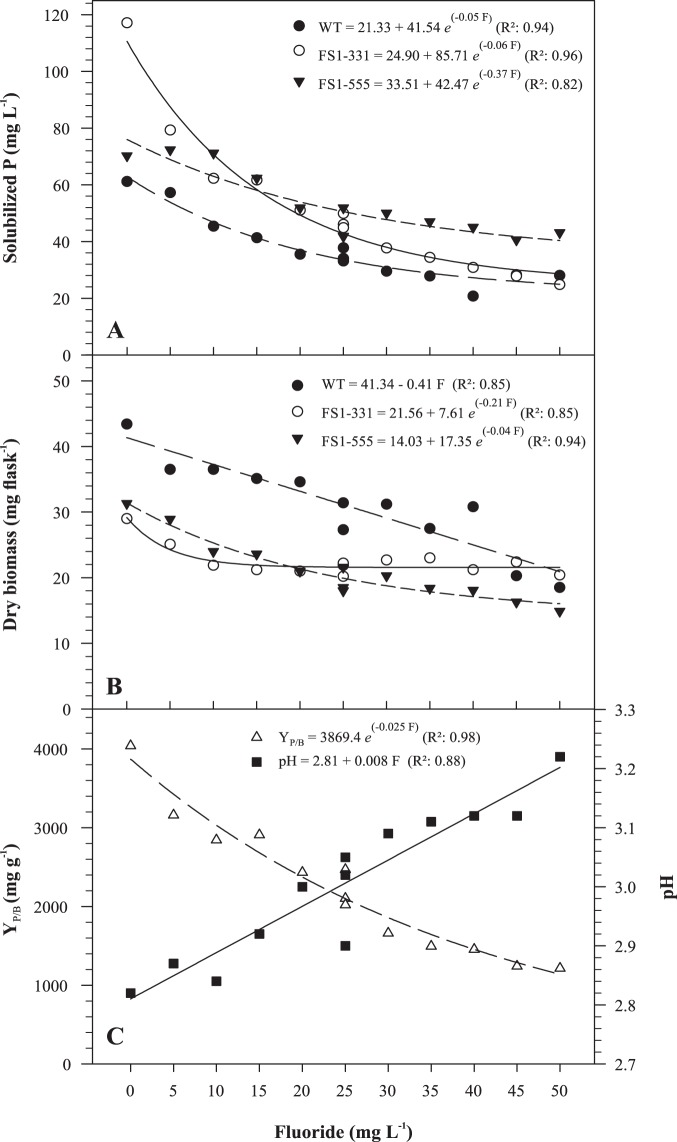
Effect of fluoride on rock phosphate solubilization by *Aspergillus niger* FS1 mutants and the wild type. (A) Solubilized P; (B) dry biomass; (C) P/biomass yield (Y_P/B_ = mg solubilized P per g of biomass) and medium pH for the mutant FS1-331. The strains were grown in NBRIP for 60 h at 32°C and 160 rpm. All regression coefficients are significant as determined by *t* test (*P*<0.01).

The selected positive mutants for Araxá RP solubilization (FS1-331 and FS1-555) were grown in media containing increasing F^−^ concentrations to simulate the release of F^−^ from Araxá RP and its effect on P solubilization by the mutants compared to the WT. The mutant FS1-331 showed higher capacity of P solubilization at low F^−^ doses, solubilizing up to 90% more P than the WT (Fig. 2a). The mutant FS1-555 was the most efficient P solubilizer at higher F^−^ doses. In general, biomass production of the mutants was lower than that of the WT (Fig. 2b). The biomass production of FS1-331 was less affected by increasing F^−^ dose (Fig. 2b) and its Y_P/B_ was reduced at higher F^−^ doses (Fig. 2c).

Higher pH values were observed in response to increasing F^−^ doses in the growth medium inoculated with FS1-331. For FS1-555, the Y_P/B_ was not affected, but the Y_P/B_ was higher than that of the WT for all doses evaluated (data not shown).

### Solubilization of different P sources by *A. niger* mutants

**Figure 3:**
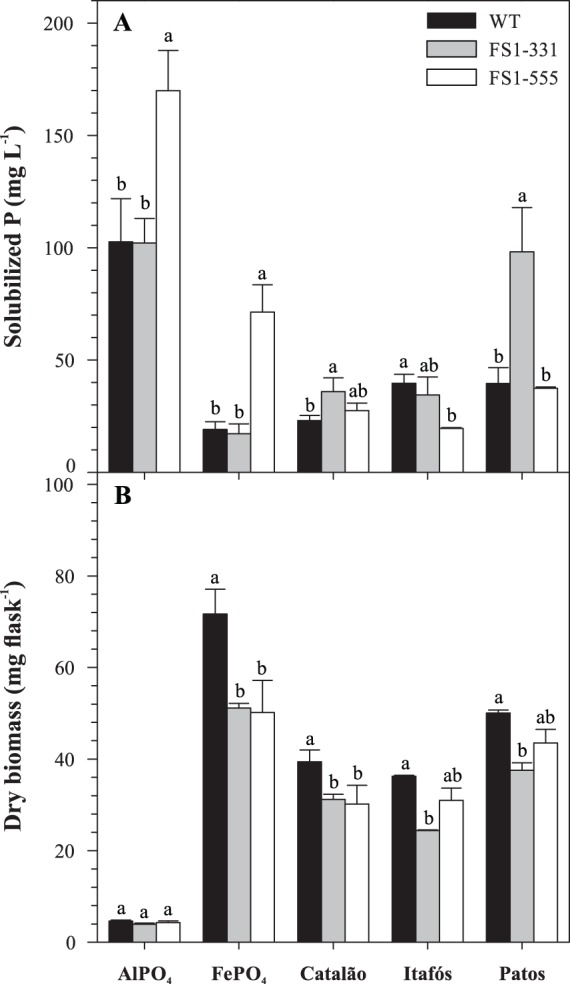
Solubilization of different P sources (A) and biomass produced (B) by *Aspergillus niger* FS1 mutants and the wild type grown in NBRIP during 60 h at 32°C and 160 rpm. For each P source, columns with the same letter are not significantly different by the Tukey’s test (*P*<0.05). Error bars represent the mean standard deviation (n = 3).

The mutant FS1-555 solubilized the highest amounts of P from pure P sources, namely AlPO_4_ and FePO_4_ (Fig. 3a). When compared to the WT, this mutant increased the concentration of solubilized P from AlPO_4_ and FePO_4_ 1.7 and 3.7 times, respectively. In the media with the RPs, FS1-331 showed the highest P-solubilizing ability, increasing the solubilization of Catalão RP by 55% and that of Patos de Minas RP by 150% in comparison to the WT. However, none of the mutants was more efficient than the WT in the solubilization of Itafós RP. Finally, the mutants produced similar amounts of biomass for all P sources (Fig. 3b).

## Discussion

Mutagenesis using UV light allowed the generation of strains with increased P-solubilizing ability in the presence of F^−^. The most prominent phenotypic difference between the mutants and the WT was the profile of organic acids produced (Fig. 1, Table 4). Organic acids are effective agents in mobilizing P from RPs or soil particles due to their capacity to form chelates with cations linked to P in poorly soluble forms [26], [27]. However, the type of organic acids produced in a microbial solubilization system is of great importance, given that the effectiveness of an organic acid as a chelating agent is highly dependent on the chemical structure, type, and position of the carboxyl and hydroxyl groups in the molecule [27], [28].

The mutants FS1-331 and FS1-555 were the only ones that produced detectable quantities of oxalic acid in the medium with Araxá RP (Table 4). The capacity to produce this acid under such conditions is probably one of the features that confer the superiority of these mutants over the WT at RP solubilization, since the production of gluconic and citric acids by the mutants was not higher than that of the WT. Previous works with the starting strain FS1 have already suggested the importance of oxalic acid for P solubilization [3], [16]. The lack of oxalic acid production by the WT in the present work is probably a consequence of the short incubation time adopted, which, in turn, highlights the efficiency of the mutants in producing this acid. Oxalic acid was reported as one of the most effective organic acid in releasing P from RPs [27]. The chemical structure of oxalic acid (C_2_H_2_O_4_) is formed by the linkage of two carboxyl groups. The proximity of these carboxyl groups increases its chelation ability [29]. Additionally, oxalate has a high tendency to precipitate with Ca^2+^, favoring the solubilization of apatite RPs [28]. Nonetheless, there must be other factors besides oxalic acid production that are related to the superiority of the mutants. Depending on the chemical composition of RPs, another organic acid, e.g. citric acid, can be more effective in solubilizing P [27]. Moreover, chemical elements released from RP can modulate the metabolism of each fungal strain [30], which could explain the differences between mutants in solubilizing different P sources (Fig. 3a). Finally, increased tolerance to F^−^ is another feature that probably improved the performance of the mutants.

As expected, mutagenesis changed the response of the fungi to F^−^. All mutants grew more than the WT in the medium with soluble P supplemented with F^−^ (Fig. 1c). These data suggest that mutagenesis allowed the isolation of mutants that were more tolerant to F^−^, considering that decreases in fungal growth are one of the major effects of this ion [4], [31]. However, in the medium with Ca_3_(PO_4_)_2_+ F^−^ the mutants grew less than the WT (Table 3). The Ca^2+^ ions released from Ca_3_(PO_4_)_2_ can react with F^−^ to form a low-solubility complex (CaF_2_) [32] which may partially alleviate F^−^ toxicity. This would permit higher growth of the WT, as already observed under low F^−^ (Fig. 1c, 2b). The negative mutant FS1-375 showed significant decreases in P solubilization in the presence of all the P sources tested (Table 3). This result can be due to a decreased organic acid production, especially citric and oxalic acids (Fig. 1, Table 4), suggesting that this mutant was the most sensitive to F^−^. The production of citric acid during RP solubilization is almost completely inhibited by F^−^ [4]. Moreover, F^−^ has antimicrobial action and can alter numerous cellular processes, such as respiration, metabolism, ion transport, secretion, endocytosis, and gene expression [14], [33].

The mutants FS1-331 and FS1-555 were more effective than the WT at solubilizing Araxá RP even at increased F^−^ doses (Fig. 2a). However, P solubilization by FS1-331 decreased sharply with increasing F^−^ doses. At higher doses, this mutant solubilized less than FS1-555, indicating that the latter is more tolerant to F^−^. This can be also observed in the medium with Ca_3_(PO_4_)_2_+ F^−^, where the FS1-555 solubilized more P than the FS1-331 and the WT (Table 3). In the case of the FS1-555 and the WT, the decreases in P solubilization can be associated to the toxic effects of F^−^ on fungal growth (Fig. 2b). However, above 10 mg L^−1^ of F^−^ there was no further decrease in biomass production by FS1-331, while the Y_P/B_ of this mutant decreased with increasing F^−^ doses (Fig. 2c). These data show that the biomass became less efficient at P solubilization probably because of deleterious effects of F^−^ on metabolic processes involved in P solubilization, i.e. production of organic acids (Table 4) and release of H^+^ resulting from cellular respiration and/or NH_4_^+^ assimilation [7], as evidenced by the higher pH observed at higher F^−^ doses (Fig. 2c).

In the medium containing the soluble P sources, higher amounts of oxalic acid were detected for FS1-331 and FS1-555 (Fig. 1a). Oxalic acid accumulation by *A. niger* is stimulated by the addition of P into the medium [34]. This characteristic is very interesting for microbial solubilization systems based on batch culture, where the concentration of soluble P increases along the time. Conversely, the synthesis of citric and oxalic acids in all treatments with soluble P supplemented with F^−^ was inhibited (Fig. 1b). Fluoride inhibits the glycolytic pathway and the Krebs cycle through binding to the active center of the enzymes of these pathways [14]. Enolase is inhibited by F^−^ [31] and, thus, in the medium with F^−^, a decrease in the pyruvate pool, an important precursor for the synthesis of citric and oxalic acids [35], may have inhibited the production of these acids. However, in the medium with Ca_3_(PO_4_)_2_ the addition of F^−^ had less effect on citric acid production (Table 4). As discussed above, F^−^ toxicity can be alleviated by formation of CaF_2_.

Gluconic acid was produced under all experimental conditions and presented little variation among the strains (Fig. 1, Table 4). In general, the presence of F^−^ is not inhibitory for gluconic acid production [4]. Gluconic acid has been found in various solubilization systems and contributes mainly with protons for the solubilization reaction [11], [36]. It seems that the increased production of gluconic acid in the medium with Ca_3_(PO_4_)_2_ supplemented with F^−^ (Table 4) was the reason for the lower pH and, consequently, the higher levels of solubilized P (Table 3) observed for FS1-555.

When the mutants were tested in different P sources, FS1-331 was more effective at solubilizing RPs (Catalão and Patos de Minas), while FS1-555 was more effective in the media with pure synthetic sources (AlPO_4_ and FePO_4_) (Fig. 3). FS1-555 possesses important characteristics for P solubilization, such as high production of citric and oxalic acids (Fig. 1a). However, this mutant seems to be more sensitive to other elements released from RP. Further studies should be done to elucidate this point.

In this work, *A. niger* mutants with improved P-solubilizing activity and higher tolerance to F^−^ were obtained using UV light-induced mutagenesis. These mutants also presented increased production of oxalic acid. Given the effectiveness of oxalic acid to solubilize apatite RPs and that most RPs are rich in F^−^, the mutants obtained, especially FS1-331, represent a significant improvement and possess a high potential for application in solubilization systems with fluoride-rich phosphate sources. Direct inoculation of these mutants in the soil-plant environment is also a prospect. However, some factors should be studied to accomplish this, such as the competition ability of the mutants against the indigenous community and the selection of vehicles for inoculation of fungal propagules into the soil.
